# Heme Oxygenase-1 Attenuates Hypoxia-Induced sFlt-1 and Oxidative Stress in Placental Villi through Its Metabolic Products CO and Bilirubin

**DOI:** 10.1155/2012/486053

**Published:** 2011-12-13

**Authors:** Eric M. George, Drew Colson, Jeremy Dixon, Ana C. Palei, Joey P. Granger

**Affiliations:** Department of Physiology and Biophysics and Center for Excellence in Cardiovascular-Renal Research, The University of Mississippi Medical Center, Jackson, MS 39216, USA

## Abstract

One of the most prevalent complications of pregnancy is preeclampsia, a hypertensive disorder which is a leading cause of maternal and perinatal morbidity and premature birth with no effective pharmacological intervention. While the underlying cause is unclear, it is believed that placental ischemia/hypoxia induces the release of factors into the maternal vasculature and lead to widespread maternal endothelial dysfunction. Recently, HO-1 has been shown to downregulate two of these factors, reactive oxygen species and sFlt-1, and we have reported that HO-1 induction attenuates many of the pathological factors of placental ischemia experimentally. Here, we have examined the direct effect of HO-1 and its bioactive metabolites on hypoxia-induced changes in superoxide and sFlt-1 in placental vascular explants and showed that HO-1 and its metabolites attenuate the production of both factors in this system. These findings suggest that the HO-1 pathway may be a promising therapeutic approach for the treatment of preeclampsia.

## 1. Introduction

Preeclampsia is a pregnancy-specific hypertensive disorder classically characterized by proteinuria and edema after the twentieth week of gestation [1]. Preeclampsia is a common complication of pregnancy, with an incidence of ~5–10% of all pregnancies [2]. Though the underlying mechanisms of preeclampsia are not well understood, a central factor believed to be important in the development of the disease is placental ischemia and hypoxia which result from a failure of the maternal uterine vasculature to remodel into high-capacitance vessels, leading to placental hypoperfusion [3–5]. In response to hypoxia, the placenta begins to produce a number of soluble factors which are secreted into the maternal circulation. Once, in circulation, these factors induce widespread maternal endothelial dysfunction, one of the key hallmarks of this disorder [6, 7]. There are a number of molecular pathways which are involved in the cascade from placental ischemia to maternal endothelial dysfunction. Two pathways which have been the subject of intense research are the production of the soluble form of the vascular endothelial growth factor (VEGF) receptor-denominated soluble fms-like tyrosine kinase (sFlt-1) and the production of placental hypoxia-induced reactive oxygen species (ROS).

VEGF signaling is necessary for endothelial health and maintenance [8]. When, in circulation, sFlt-1 binds to free VEGF and placental growth factor (PlGF) sequestering them and making them unavailable for proper signaling [9]. sFlt-1 can be directly induced by hypoxia through the actions of HIF-1*α* and has been shown to be produced by human placental trophoblasts and villi in response to decreased oxygen [10–12]. It has also been shown to be produced in the placenta during preeclampsia and is found to be elevated in the circulation of preeclamptic women, often before the onset of maternal symptoms [13, 14]. In addition, numerous experimental models have demonstrated the importance of sFlt-1 overexpression in the development of preeclampsia [15–18]. A second factor implicated in the pathophysiology of preeclampsia is oxidative stress. Oxidative stress has been shown to be elevated in both the placenta and the maternal vasculature of women with preeclampsia [19–21]. The symptoms associated with experimental rodent placental hypoperfusion have also shown to be attenuated by either the superoxide dismutase mimetic Tempol or the NADPH oxidase inhibitor apocynin [22, 23]. This suggests that oxidative stress, at least partially induced by NADPH oxidase, is a crucial factor in the symptomatic manifestation of preeclampsia.

One potential palliative agent suggested for the normalization of these two pathways is the enzyme heme oxygenase-1 (HO-1). HO-1 typically catalyzes the rate-limiting step in the heme salvage pathway, converting the prooxidant heme to biliverdin, which is then rapidly converted by biliverdin reductase to bilirubin, a known antioxidant [24, 25]. As a side product, HO-1 releases carbon monoxide, a vasodilator [26]. Recently, it has been reported that HO-1 could negatively regulate VEGF or interferon-*γ*-induced sFlt1- release in vitro. Furthermore, CO could directly act in a similar manner [27]. Also, we have recently demonstrated that induction of HO-1 in a rodent placental ischemia model attenuated the associated hypertension and angiogenic imbalance [28]. In the present work, we have extended this previous work to examine the direct effects of HO-1 and its byproducts CO and bilirubin on hypoxia-induced oxidative stress and sFlt-1 production.

## 2. Materials and Methods

### 2.1. Animals

Timed pregnant Sprague-Dawley rats were obtained from Harlan, Inc. (Indianapolis, Ind). All animal protocols were approved by The University of Mississippi Medical Center Institutional Animal Care and Use Committee and followed the National Institutes of Health Guidelines for the Care and Use of Laboratory Animals. Animals were maintained at constant temperature (23°C) with a 12 : 12 h light : dark cycle. On day 19 of gestation, the animals were sacrificed by pneumothorax and cardiac excision followed by tissue harvest.

### 2.2. Placental Explants

Placental explants were cultured as described previously [29]. Briefly, after tissue excision, placentas were immediately placed into cold Dulbecco's Phosphate Buffered Saline (Sigma, St. Louis, Mo). The mesometrium and decidua were carefully removed, and villous bundles from the trophospongium and labyrinth were excised. The villous explants were plated in 24-well cell culture plates coated with 0.2 mL of Matrigel Matrix Basement Membrane from BD Bioscience (Bedford, Mass). Explants were grown in Dulbecco's Modified Eagle's Media-Ham's F-12 supplemented with 10% FBS, 100 *μ*g/mL streptomycin, 100 U/mL penicillin, and 25 *μ*g/mL ascorbic acid as previously described [30]. The explants were maintained at constant oxygen tensions of either 6% or 1% in double gas incubators purged with nitrogen.

### 2.3. Experimental Protocol

Explants were randomly assigned into control and experimental groups. Control explants were incubated in media with no supplementation. The HO-1 inducer cobalt (III) protoporphyrin IX chloride (CoPP, Frontier Scientific, Logan, Utah) was prepared in 0.1 M NaOH in saline, and the pH was adjusted to a final pH of 8.5 CoPP was utilized at a final concentration in culture of 20 *μ*M. CORM-3 has been previously described [31] and was prepared in media immediately before use at a final concentration of 40 *μ*M. Bilirubin was utilized at a final concentration of 100 *μ*M. At 48 hours after treatment, the cell culture media was removed from the explants and both media and tissue were frozen for further analysis. A minimum of 5 samples were obtained for every experimental group.

### 2.4. Western Blotting

Total intracellular protein was extracted by 5X repeated freeze-thaw lysis in FT buffer (600 mM KCl, 20 mM Tris-Cl, pH 7.8, 20% glycerol, 0.4 mg/mL Pefabloc, 10 *μ*g/mL leupeptin, 10 *μ*g/mL pepstatin, and 5 *μ*g/mL aprotinin) [32]. Protein concentration was determined by Bradford assay (Bio-Rad). For western blots, 30 *μ*g of protein was subjected to SDS-PAGE on 4–20% gradient SDS-polyacrylamide gels (Bio-Rad). Membranes were blocked with Odyssey blocking buffer (LI-COR, Lincoln, Neb) for two hours at room temperature. Primary incubation was undertaken overnight at 4°C with a rabbit anti-HO-1 polyclonal antibody (StressGen, Vancouver, Calif) at 1 : 2000 and a mouse anti-*β*-actin antibody (Gentest) at 1 : 5000. Secondary antibody incubation was done with Alexa Fluor 680 goat anti-rabbit (Molecular Probes) and IRDye 800 goat anti-mouse IgG (Rockland) for one hour at room temperature. Fluorescence was detected on an Odyssey infrared imager (LI-COR) for simultaneous detection of both species. Blot analysis was performed with the supplied Odyssey software, and HO-1 was normalized to *β*-actin, with *n* = 6 in each group.

### 2.5. Measurement of Superoxide by DHE

DHE fluorescence assays were carried out as previously described [33]. Individual wells of a black 96-well microtiter plate were filled with 200 uL of 5 *μ*M DHE (Invitrogen) diluted into phosphate buffered saline. Fluorescence was monitored by excitation at 510 nm and emission at 610 nm. Fluorescence was monitored every 2 minutes for a total of one hour and the resulting measurements averaged over the life of the experiment. For each group, *n* = 7–13. Fluorescence was normalized to the average of normoxic controls. Statistical comparisons were performed by one-way ANOVA.

### 2.6. sFlt-1 Measurement

Total protein concentration of the cell culture supernatants was determined by the Bicinchoninic Acid (BCA) assay (Pierce, Rockford, Ill) using bovine serum albumin (BSA) as a standard. Measurement of s-Flt1 was performed by sandwich ELISAs (R&D Systems, Minneapolis, Minn) according to manufacturer protocols. The plates were read on a Tecan GENios microplate reader, and quantitation was performed with Megellan version 4.1 software. sFlt-1 levels were normalized to the total amount of media protein, and the results graphed with Origin Pro 8 (Microcal), which was also used for all statistical analyses. Statistical significance was determined by one-way ANOVA, with a significance threshold of *P* < 0.05.

## 3. Results

### 3.1. Hypoxia Suppresses HO-1 Expression in Placental Villi and CoPP Treatment Restores Normal HO-1 Levels

**Figure 1:**
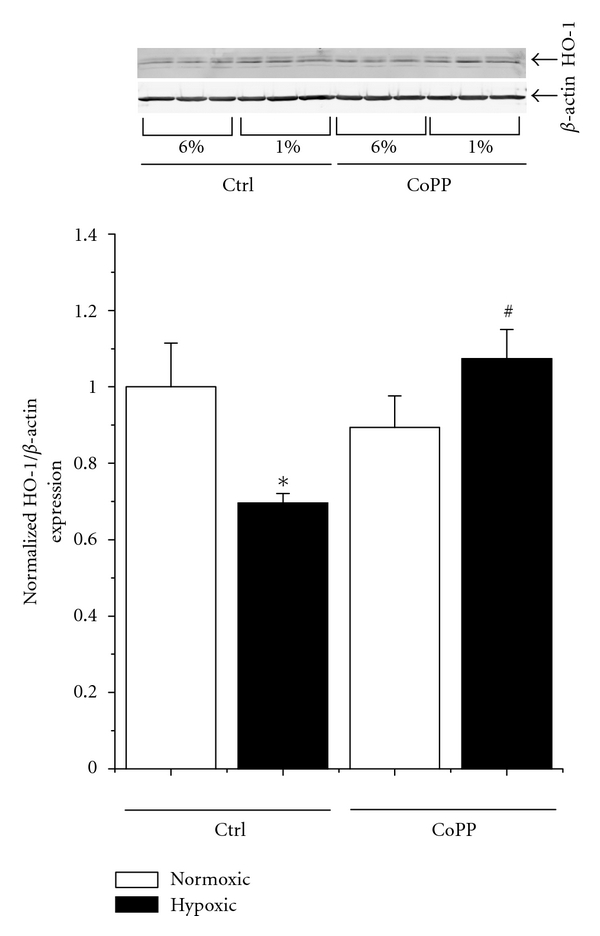
*Hypoxia decreases HO-1 expression in placental villous explants, which can be restored by CoPP. *Placental villous explants were incubated for 48 hours under 6% (normoxic) or 1% (hypoxic) oxygen to simulate the oxygen tension in a health and preeclamptic placenta. As assayed by western blot normalized to *β*-actin, in response to hypoxia treatment, there was an approximate 30% reduction in the tissue levels of HO-1. Cobalt protoporphyrin (CoPP), while having no significant effect under hypoxic conditions, fully restored HO-1 expression in the hypoxic villi. Statistical significance at the *P* < 0.05 level is indicated by “∗” versus normoxic controls and “#” versus hypoxic controls.

In order to assess the effect of hypoxia and CoPP on HO-1 expression in placental villi, villous explants were excised from the trophospongium and labyrinth of rodent placentas on day 19 of gestation. These tissues were cultured on synthetic basement membrane in either 6% (normoxic) or 1% (hypoxic) oxygen, reflecting the approximate oxygen tension of a normal and hypoxic placenta, respectively. As can be seen in Figure 1, at 48 hours, villous bundles in the hypoxic exhibited an approximate 30% reduction in their *β*-actin normalized HO-1 expression (6% = 1 ± 0.11 versus 1% = 0.69 ± 0.02, *P* < 0.05). In response to CoPP, there was no significant difference in the expression of HO-1 in the 6% treated group (0.89 ± 0.08). When CoPP was administered to villi exposed to 1% oxygen, however, there was a significant normalization of HO-1 expression when compared to hypoxic controls (1% = 0.69 ± 0.02 versus 1% + CoPP = 1.07 ± 0.08, *P* < 0.05), indicating that CoPP can restore HO-1 expression in hypoxic villi.

### 3.2. HO-1 Induction and the HO-1 Byproduct Bilirubin Attenuate Hypoxia-Induced Superoxide in Placental Villous Bundles

**Figure 2:**
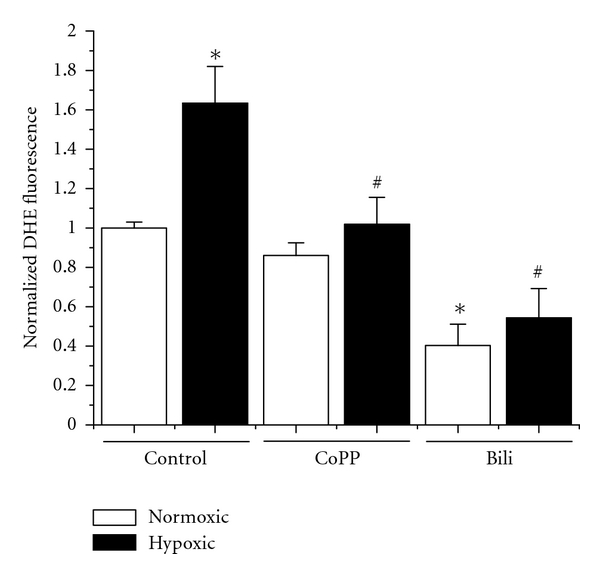
*HO-1 and bilirubin suppress hypoxia-induced increases in superoxide production. *In response to culture in hypoxic conditions, there is a significant ~60% increase in the level of superoxide generated by the placental explants as assayed by dihydroxyethidium (DHE) fluorescence. Administration of CoPP had no significant effect on normoxic explants but completely attenuated the hypoxia-induced increase seen in the control explants. Exogenous application of bilirubin (Bili) had a significant attenuation of superoxide in both the normoxic and hypoxic samples when compared to their respective controls. Statistical significance at the *P* < 0.05 level is indicated by “∗” versus normoxic controls and “#” versus hypoxic controls.

We next assessed the effects of both HO-1 levels and the direct effect of the HO-1 product bilirubin, a known antioxidant, on hypoxia-induced superoxide production. As seen in Figure 2, in response to hypoxic exposure, there is a significant increase in the tissue levels of superoxide of approximately 60% as determined by dihydroethidium fluorescence (6% = 1 ± 0.03 versus 1% = 1.63 ± 0.19, *P* < 0.05). Administration of CoPP had no significant difference under normoxic conditions (0.86 ± 0.06), but hypoxic tissues treated with CoPP exhibited a marked decrease in DHE fluorescence when compared to hypoxic controls (1% = 1.63 ± 0.19 versus 1% + CoPP = 1.01 ± 0.14, *P* < 0.05). Incubation with bilirubin had a marked effect on detected superoxide in both normoxic and hypoxic tissues. Under 6% oxygen, there was a significant decrease in DHE fluorescence when compared to normoxic controls (6% = 1 ± 0.03 versus 6% + Bili = 0.040 ± 0.11, *P* < 0.05). A similar level of DHE fluorescence was seen in hypoxic tissues, which were also significantly different from their hypoxic controls (1% = 1.63 ± 0.19 versus 1% + Bili = 0.54 ± 0.15, *P* < 0.05). These data suggest that both HO-1 and bilirubin significantly attenuate hypoxia-induced superoxide in placental villi.

### 3.3. HO-1 and Its Byproducts Attenuate Hypoxia-Induced sFlt-1 Production in Placental Villi

**Figure 3:**
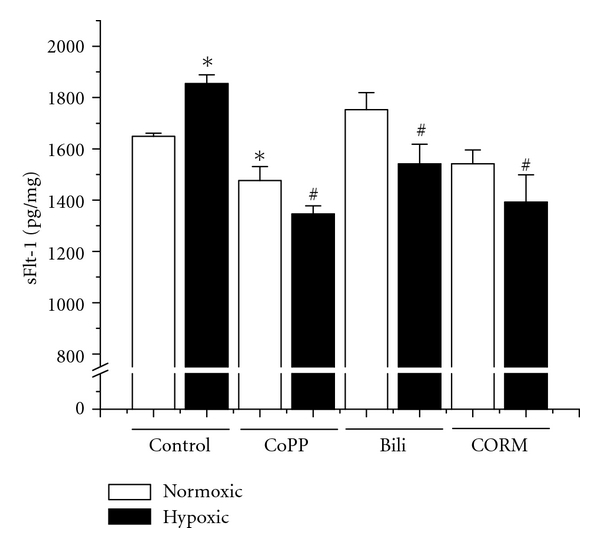
*HO-1 induction, CO, and Bilirubin attenuate hypoxia-induced sFlt-1 secretion.* In response to hypoxic incubation, placental explants demonstrate a significant increase in the production of sFlt-1 when compared to normoxic controls. Administration of CoPP significantly attenuated sFlt-1 released under both normoxic and hypoxic conditions. Bilirubin, while having no effect under normoxic conditions, slightly but significantly decreased sFlt-1 release when compared to hypoxic controls. Administration of a CO-releasing molecule (CORM) again had no effect under normoxic conditions but significantly attenuated sFlt-1 release in hypoxic explants. Statistical significance at the *P* < 0.05 level is indicated by “∗” versus normoxic controls and “#” versus hypoxic controls.

We also wished to determine what effect HO-1 and its byproducts would have on the hypoxia-induced production of sFlt-1. As seen in Figure 3, in response to hypoxia treatment, secreted sFlt-1 is significantly increased in response to hypoxia exposure (6% = 1649 ± 12.1 pg/mg versus 1% = 1854 ± 35 pg/mg, *P* < 0.05). Administration of CoPP decreased the amount of sFlt-1 secreted under both normoxic (6% = 1649 ± 12.1 pg/mg versus 6% + CoPP = 1477 ± 54 pg/mg, *P* < 0.05) and hypoxic (1% = 1854 ± 35 pg/mg versus 1349 ± 32 pg/mg, *P* < 0.05) conditions when compared to their respective controls. While bilirubin had no significant effect on sFlt-1 under normoxic conditions (1753 ± 56 pg/mg), in hypoxia-exposed explants, there was a significant decrease in the production of sFlt-1 when compared to hypoxic controls (1% = 1854 ± 35 pg/mg versus 1542 ± 76 pg/mg, *P* < 0.05). Similarly, administration of a CO donor molecule had no significant effect on the production of sFlt-1 under normoxic conditions (6% = 1649 ± 12.1 pg/mg versus 6% + CORM = 1542 ± 53 pg/mg) but exhibited a marked attenuation of sFlt-1 under hypoxic conditions (1% = 1854 ± 35 pg/mg versus 1392 ± 107 pg/mg, *P* < 0.05). Collectively, these data suggest that HO-1 through both CO and bilirubin suppresses hypoxia-induced sFlt-1 in placental villi.

## 4. Discussion

Preeclampsia remains a major health concern, affecting at least one out of every twenty pregnancies [34]. Besides the immediate risk for mother and fetus, there is increasing evidence that preeclampsia confers increased risk for cardiovascular complications to the offspring in later life [35]. One of the major roadblocks in the management of preeclampsia is the lack of an effective pharmacological intervention for its treatment. A potential therapy which has been proven effective in numerous experimental forms of hypertension is the manipulation of the HO-1 pathway [36–39].

HO-1 is hypothesized to attenuate hypertension through multiple pathways. HO-1 produces two bioactive compounds as products of heme metabolism: CO and bilirubin. Bilirubin has been shown in numerous systems to function as a powerful antioxidant [24, 25]. As it is recognized that production of reactive oxygen species is a major component of varied forms of hypertension, moderate increases in bilirubin could act to decrease overall oxidative stress. CO acts as a potent vasodilator, functioning in a manner similar to endothelium-derived relaxing factor [26]. Of particular interest to preeclampsia, HO-1 and CO have been shown to directly inhibit production of VEGF and interferon-*γ*-induced secretion of sFlt-1 [27]. Additionally, it has been reported that CO derived from HO-1 in vascular smooth muscle could inhibit production of endothelin-1 [40], a protein shown to be a final common pathological factor in several experimental models of preeclampsia.

We have recently demonstrated that induction of the HO-1 pathway could significantly attenuate the preeclampsia-like pathological manifestations associated with experimental placental ischemia in the rodent. Specifically, HO-1 attenuated the associated hypertension, with concomitant decreases in placental sFlt-1 and oxidative stress, and increased circulating bioavailable VEGF [28]. However, in this in vivo model, it was not possible to determine the direct effects of HO-1 and the individual metabolites on the regulation of each of these factors within the placenta. In the present work, we have utilized a previously established model of placental vascular bundle culturing to determine the importance of each of these factors in the regulation of oxidative stress and sFlt-1 in response to hypoxia.

Western blotting analysis demonstrated that hypoxia exposure significantly decreased the expression of HO-1 in the vascular bundles. This is surprising, as numerous reports have indicated hypoxia induces an increase in HO-1 [41–43]. However, this effect has never been examined in the placental vasculature. This could help explain the increase in oxidative stress in the preeclamptic placenta. CoPP administration had no effect under normoxic conditions but in hypoxia restored HO-1 to normoxic levels. This is consistent with our previous data which shows that CoPP induces HO-1 in the placenta only with the secondary insult of placental ischemia/hypoxia [28].

In response to hypoxia exposure, the placental explants exhibited increased production of sFlt-1. CoPP administration significantly decreased sFlt-1 secretion to levels which were below even normoxic controls. Though CO has been shown to negatively regulate VEGF and interferon-*γ*-induced sFlt-1 in placental explants [27], its effect on hypoxia-induced sFlt-1 release has not been reported. As suggested in the literature, CO did indeed significantly attenuate hypoxia-induced sFlt-1, returning to levels equivalent to CoPP induction. Perhaps most surprisingly, administration of bilirubin also significantly decreased the secretion of sFlt-1 during hypoxia. This may suggest that oxidative stress is playing a role in the induction of sFlt-1, though the s ameliorative effect of bilirubin was not as great as either CoPP or CO, suggesting that CO is the major factor in HO-1's effect on sFlt-1.

Superoxide was also increased in the explants exposed to hypoxia when compared to normoxic controls. Treatment with CoPP, which does not induce HO-1 expression under normoxic conditions, had no effect on superoxide levels. However, under hypoxic conditions where CoPP normalizes HO-1 levels, there was a significant reduction in the amount of superoxide produced by the explants. Tellingly, the exogenous application of bilirubin to the explants under either normoxic or hypoxic conditions led to a significant decrease in superoxide when compared to their respective controls. This is perhaps an intuitive result given the known antioxidant properties of bilirubin but strongly suggests that moderate elevations in bilirubin production as a result of increased HO-1 are driving the reduction in oxidative stress. This demonstrates a second pathway through which HO-1 induction is directly affecting hypoxia-induced changes in the placental vasculature. It seems evident from these data as a whole, that HO-1 induction, through both CO and bilirubin production, is having a direct effect on the placental vasculature during ischemia/hypoxia. Continued studies into the safety and efficacy of agents to increase HO-1 or deliver these metabolites directly is warranted.

## 5. Conclusions

We have demonstrated here that induction of HO-1, or delivery of its bioactive metabolites CO and bilirubin, is capable of significantly downregulating two major hypoxia-induced factors (oxidative stress and sFlt-1) implicated in the etiology of preeclampsia. This suggests a promising therapeutic approach for the treatment of preeclampsia. Future studies both in vitro and in vivo are necessary to fully elucidate the therapeutic potential of this approach.
